# Seeing tobacco mosaic virus through direct electron detectors

**DOI:** 10.1016/j.jsb.2014.12.002

**Published:** 2015-02

**Authors:** Simon A. Fromm, Tanmay A.M. Bharat, Arjen J. Jakobi, Wim J.H. Hagen, Carsten Sachse

**Affiliations:** a EMBL – European Molecular Biology Laboratory, Structural and Computational Biology Unit, Meyerhofstr. 1, 69117 Heidelberg, Germany; b MRC Laboratory of Molecular Biology, Structural Studies Division, Francis Crick Avenue, Cambridge CB2 0QH, UK; c EMBL – European Molecular Biology Laboratory, Hamburg Unit, Notkestrasse 85, 22603 Hamburg, Germany

**Keywords:** Tobacco mosaic virus, Direct electron detectors, High-resolution electron cryo-microscopy, Single-particle helical reconstruction, Radiation damage

## Abstract

With the introduction of direct electron detectors (DED) to the field of electron cryo-microscopy, a wave of atomic-resolution structures has become available. As the new detectors still require comparative characterization, we have used tobacco mosaic virus (TMV) as a test specimen to study the quality of 3D image reconstructions from data recorded on the two direct electron detector cameras, K2 Summit and Falcon II. Using DED movie frames, we explored related image-processing aspects and compared the performance of micrograph-based and segment-based motion correction approaches. In addition, we investigated the effect of dose deposition on the atomic-resolution structure of TMV and show that radiation damage affects negative carboxyl chains first in a side-chain specific manner. Finally, using 450,000 asymmetric units and limiting the effects of radiation damage, we determined a high-resolution cryo-EM map at 3.35 Å resolution. Here, we provide a comparative case study of highly ordered TMV recorded on different direct electron detectors to establish recording and processing conditions that enable structure determination up to 3.2 Å in resolution using cryo-EM.

## 1 Introduction

The recent introduction of direct electron detectors (DEDs) provide new and exciting possibilities to the field of three-dimensional (3D) electron cryo-microscopy (cryo-EM) (Grigorieff, 2013). When biological samples are rapidly plunge-frozen in a thin layer of vitreous ice using liquid ethane they can be studied in their native structural state (Adrian et al., 1984). Until recently, near-atomic resolution insights have only been possible for molecules that are arranged symmetrically such as two-dimensional crystals, helical arrays or icosahedral assemblies (Henderson et al., 1990; Unwin, 2005; Yu et al., 2008; Zhang et al., 2008). Recent hardware developments have resulted in new DED devices that enabled atomic-resolution single-particle structures of specimens such as the 20S proteasome with lower order symmetry (D7) or the mitochondrial ribosome with no symmetry at all (Amunts et al., 2014; Li et al., 2013a). Resolutions below 3.5 Å allow *de novo* building of atomic models and provide unprecedented insights into the molecular function of biological macromolecules (Allegretti et al., 2014; Amunts et al., 2014; Liao et al., 2013; Voorhees et al., 2014).

The main architectural advantage of new-generation detectors over previously used phosphor-fiber optics CCD cameras or photographic film is the capability to directly capture electrons, thus omitting the intermediate step of signal-to-light conversion (Faruqi and Henderson, 2007). Hence, the latest generation of DEDs has surpassed the performance of film as a high-resolution recording medium. Furthermore, the read-out performance is significantly faster and thus it has become possible to record micrographs in ‘movie mode’ where multiple frames constitute the traditional micrograph recorded from a single exposure. This feature has opened new means to track individual particles in frames over time and has led to new insights on beam-induced movement (Brilot et al., 2012). Furthermore, frame processing can compensate for beam-induced movement and thus improve the resolution of 3D reconstructions from DEDs (Bai et al., 2013; Campbell et al., 2012; Li et al., 2013a). Currently, five DEDs are commercially available: the Gatan K2 Summit, the FEI Falcon I and II, and the Direct Electron DE-12 and DE-20. The K2 Summit is the only camera that can be operated as an electron-counting device to detect electrons at sub-pixel accuracy in super-resolution mode. Grigorieff and Henderson characterized the performance of these devices by measuring their detective quantum efficiency (DQE) and modulation transfer function (MTF) curves (McMullan et al., 2014; Ruskin et al., 2013) with a focus on the most commonly available voltage of electron microscopes at 200 kV and 300 kV. While these tests provide objective characterization of information transfer, it is less clear how these measurements translate into the quality of 3D reconstructions. To address this issue, a biological test specimen of high stability is required so that few micrographs suffice to compute high-resolution 3D structures using a standard image-processing workflow.

Therefore, we turned to tobacco mosaic virus (TMV) that has been extensively studied since the first days of structural biology (Bernal and Fankuchen, 1941). Electron diffraction patterns reveal that the helical order could be measured up to 2.3 Å (Cyrklaff and Kühlbrandt, 1994). With the development of single-particle based helical reconstruction the resolution was pushed to below 5 Å (Sachse et al., 2007) using 200,000 asymmetric units recorded on film. Subsequently, TMV recorded on a Gatan US4000 CCD camera gave rise to a 4.6 Å resolution structure (Clare and Orlova, 2009). These structures could be improved to 3.3 Å by using a total of 1,900,000 asymmetric units (Ge and Zhou, 2011) recorded on film. Given the large amount of published structural data on TMV and the known high preservation of helical order, we set out to compare the performance of two commonly available DEDs by testing their effect on the quality of TMV structures determined using a previously published single-particle based helical reconstruction workflow (Desfosses et al., 2014).

In the current article, we compared the performance of the two common DED cameras, K2 Summit and Falcon II, in cryo-EM using TMV as a test specimen. When imaged in the same microscope, micrographs from both cameras give rise to ∼4 Å resolution image reconstructions of very similar quality using only 44,000 asymmetric units. A comparison between micrograph-based and segment-based motion correction revealed that both strategies equally lead to significant improvements in the resolution of the TMV reconstructions. Moreover, when investigating the effect of increasing electron dose on the TMV structure we found that radiation damage is side-chain specific and this specificity is very similar to that of X-ray crystallography experiments. Finally, using a total of 450,000 asymmetric units we determined a high-resolution cryo-EM map of TMV at 3.35 Å resolution that contains structural details up to 3.2 Å.

## 2 Results

### 2.1 Performance comparison of K2 Summit and Falcon II recorded on the same microscope

The recently published results based on DED data (Amunts et al., 2014; Bai et al., 2013; Campbell et al., 2012; Fernandez et al., 2013; Li et al., 2013a; Liao et al., 2013) were obtained using different commercially available devices. In this context, it is of urgent interest to the scientific community to objectively measure their performance and in particular to compare their direct effect on the final 3D image reconstruction of biological macromolecules. Therefore, we set out to compare the quality of TMV 3D image reconstructions from electron cryo-micrographs acquired with a Gatan K2 Summit and a FEI Falcon II detector using a recently described and publicly available standardized single-particle based 3D helical reconstruction workflow from the software suite SPRING (Desfosses et al., 2014).

When comparing DED performance with existing high-resolution 3D structures, the main concern is that the data sets were recorded on different electron microscopes. Moreover, different samples and preparation protocols are being used, so that results may not be generally conclusive about DED performance. Therefore, we recorded two small data sets from the same EM grid of TMV on the MRC-LMB Titan Krios that is equipped with both K2 Summit and Falcon II DED devices. Here, the Falcon II is in the pre-GIF position while the K2 Summit is attached to the end of a Gatan Quantum energy filter. The data was acquired with the energy slit retracted. In order to record data at comparable physical pixel sizes the exact imaging conditions in the microscope had to be varied due the different position of the cameras. We collected micrographs in movie mode with 2–3 e^−^/Å^2^ dose per frame accumulating to a total of 22 or 16 frames and 43 e^−^/Å^2^ for the K2 Summit and Falcon II camera, respectively. Optimal performance of the K2 Summit and the Falcon II requires the use of significantly different dose rates at 5–10 e^−^/pixel/s (Li et al., 2013b) vs. 50 e^−^/pixel/s, respectively. To make our studies comparable we chose the typical dose rates that were used in a number of high-resolution structure studies from the K2 and Falcon II camera (Amunts et al., 2014; Bai et al., 2013; Li et al., 2013a; Liao et al., 2013).

**Supplementary Fig. 1:**
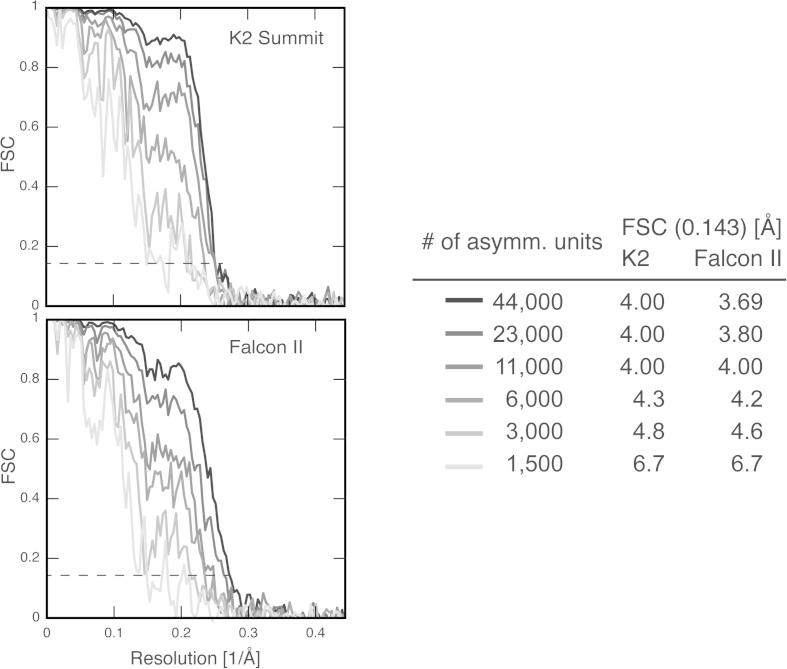
Comparison of Falcon II and K2 Summit structures computed with increasing number of asymmetric units. Left. Fourier-shell correlation curves from K2 Summit (top) and Falcon II (bottom) with increasing number of asymmetric units color-coded from gray to black. Right. Table of respective resolution cutoff values (0.143).

**Fig. 1:**
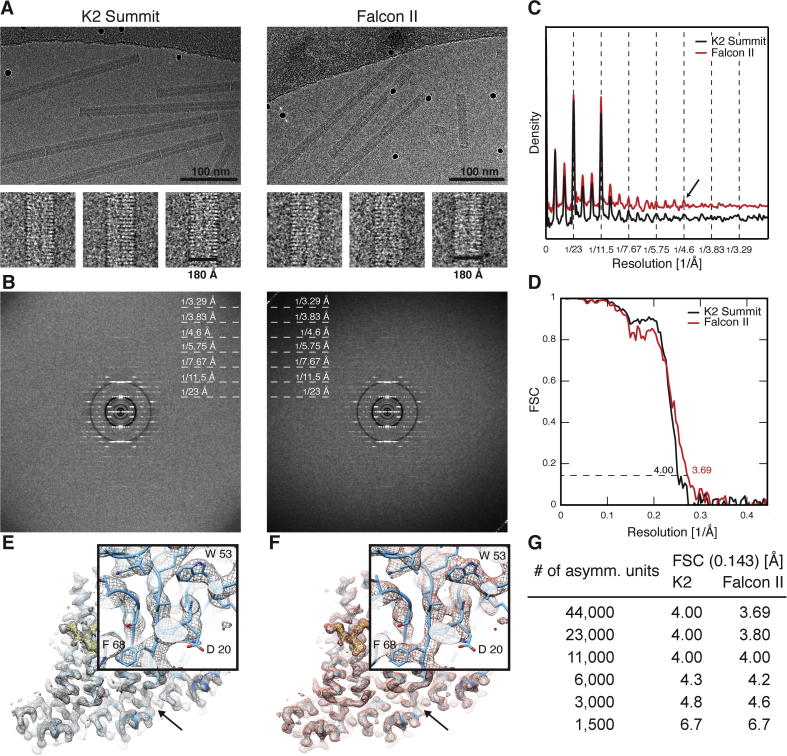
Comparison of 3D reconstructions of an intermediately sized data set from the direct electron detector K2 Summit and Falcon II recorded on the same TMV grid with the MRC-LMB FEI Titan Krios microscope. (A) Micrograph and gallery of 3 representative in-plane rotated segments of TMV recorded on K2 Summit (left) and Falcon II (right). (B) Sum of approx. 690 overlapping power spectra from K2 Summit (left) and Falcon II (right). (C) B factor-compensated and base-line corrected collapsed power spectrum of K2 Summit (black) and Falcon II (red) indicate visibility of peaks up to 4.6 Å corresponding to a meridional layer line (arrow). (D) Fourier shell correlation of the K2 Summit (black) and Falcon II data sets (red). (E/F) Respective cryo-EM maps from K2 Summit (EMD-2833) and Falcon II (EMD-2834) superimposed with refined PDB structure. Inset. Visualization of separated β-strands in twisted β-sheet at higher radius. (G) Resolution cutoffs of the K2 Summit and Falcon II structures with decreasing number of asymmetric units per reconstruction.

For the initial analysis of the raw data without subject to any structural refinement, we generated approximately 690 overlapping segments with a step size of 90 Å and applied only the in-plane rotation as determined from the interactive picking of the viruses (Fig. 1A). The sum of the power spectra from overlapping segments represents an unbiased measure of the order and the recorded information content of the helical segments (Fig. 1B). The collapsed power spectra profiles where pixel rows along the layer lines were averaged from the two data sets revealed that peaks from meridional layer lines were clearly detectable up to 4.6 Å (Fig. 1C). Using approximately 44,000 asymmetric units, we determined two cryo-EM maps at a resolution of 4.0 and 3.7 Å (FSC 0.143 cutoff) for the K2 Summit and Falcon II respectively including motion correction (see below and in Fig. 1D). The FSC curve from the K2 Summit reconstruction had slightly stronger low-resolution and weaker high-resolution signal when compared with the Falcon II curve. Visual inspection of the two maps revealed almost identical quality of the density. At this resolution, the β-strands within the small twisted β-sheet at higher outer radius position are clearly separated in both maps (Fig. 1E and F). Given the similarity in the achieved resolution, we further assessed whether one of the detectors requires significantly less data to achieve a particular resolution. Therefore, we computed six reconstructions for each data set by approximately doubling the number of asymmetric units from 1500 to 44000. The FSC resolution cutoffs showed a similar behavior for both detectors starting from 6.7 and reaching 4.0/3.7 Å (Fig. 1G). Comparison of FSC resolution curves between the K2 Summit and Falcon II detectors (Supplementary Fig. S1) confirmed that there were no significant differences in the amount of data required to achieve a particular resolution.

### 2.2 Comparison of segment-based and micrograph-based motion correction

The cryo-EM maps (Fig. 1) described above were obtained with frame processing depositing a total dose of 21.5 e^−^/Å^2^ for the Falcon II and the K2 data set. As exposures are collected in movies as an image series over time, frame processing has become an important factor to compensate for movement during the exposure and to produce maps of highest resolution (Bai et al., 2013; Campbell et al., 2012; Li et al., 2013a). In principle, two methods of motion correction have been put forward. First, in case of large icosahedral particles with high signal, the motion correction was directly based on the alignment of individual particles within a subset of frames (Campbell et al., 2012). In practice, this requires the starting parameters of the frame sum to perform a local refinement of *x*–*y* shifts and Euler angles to compensate the translational and rotational movements during the exposure. This approach has been extended with additional restraints such as running averages of frame sets on viruses and Bayesian statistics on images of ribosomal particles (Bai et al., 2013). Second, in the case of smaller particles, the motion is tracked using the signal from a field of multiple particles, i.e. the frames from larger sub-regions of the micrograph or entire micrographs (Li et al., 2013a; Scheres, 2014; Shigematsu and Sigworth, 2013). While micrograph-based motion correction accounts for *x* and *y* translation compensation it has no means of correcting possible rotational movements of the specimen.

**Fig. 2:**
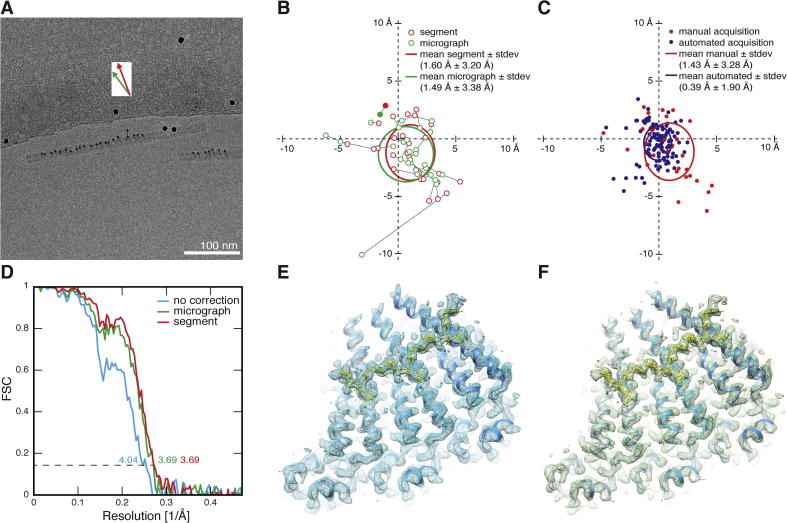
Comparison of segment-based and micrograph-based motion correction on Falcon II. (A) Micrograph with superimposed translation vectors computed from segment-based (black) motion correction (30× enlarged). White inset shows determined average shift vector of segment-based (red) and micrograph-based (green) motion correction (magnitudes are scaled to match plot in B). (B) Scatter plot of mean translation vectors between 2.4 and 21.5 e^−^/Å^2^ of all processed micrographs of the Falcon II MRC-LMB data set from segment-based (red) and micrograph-based motion correction (green). Values corresponding to a single micrograph are connected by a black line. The filled dot pair represents the micrograph shown in (A). (C) Scatter plot of mean translation vectors of Falcon II EMBL data set (blue, between 2.0 and 18.4 e^−^/Å^2^) that was collected using automated drift monitoring and MRC-LMB Falcon II data set (red, between 2.4 and 19.1 e^−^/Å^2^) that was collected manually. (D) Fourier shell correlation of no motion correction (light blue), segment-based (red) and micrograph-based (green) motion correction from data collected with Falcon II MRC-LMB. (E/F) Corresponding cryo-EM reconstructions from no correction and micrograph-based motion correction at 4.0 and 3.7 Å resolution. The map corresponding to the segment-based motion correction is shown in Fig. 1F.

In order to compare the performance of the two approaches, we processed the TMV data taken on the MRC-LMB Falcon II using segment-based and micrograph-based motion correction. Inspection of applied shifts from frame sets 1 to 8 (2.4–21.5 e^−^/Å^2^) on a single micrograph and individual segments revealed that the computed magnitude and direction of the shifts are very similar (Fig. 2A). To analyze the trend over a series of micrographs, we determined the average shift vector that was applied to all segments of a single micrograph and in addition the shift determined by micrograph-based motion correction (Fig. 2B). The distribution shows good agreement between the two motion correction methods and their determined translation vectors. The differences ranged from 0.5 to 5 Å, with a very similar mean shift and associated standard deviation of 1.6 ± 3.2 Å and 1.5 ± 3.4 Å for segment and micrograph-based motion correction, respectively. Given that the mean shift magnitude was not zero, the stage exhibited measureable drift during the acquisition. For a second larger data set taken on the EMBL Falcon II, the micrograph shifts had smaller magnitudes of 0.4 ± 1.9 Å compared with the MRC-LMB Falcon II data set (Fig. 2C). We attribute the difference in shift vectors between the MRC-LMB and EMBL microscopes to the operation mode. While the EMBL data set was collected with the drift-monitoring feature of FEI’s EPU software to <0.8 Å/s, the MRC-LMB microscope was operated manually for data acquisition without such an automatic drift monitoring. A potential advantage of the segment-based approach is that it can also compensate for rotational movements of the specimen by adjusting the Euler angles for each frame set. Subsequently, we determined the structures of the MRC-LMB Falcon II data set that has higher average drift using both motion correction strategies. It is evident from the FSC curves that motion correction does provide an improvement from 4.0 to 3.7 Å (Fig. 2D). Despite the signal decrease of the segments from frames, the estimated alignment errors increased only by a small margin (Table 1) and as a result the segment-based correction performed slightly better over micrograph-based correction although the rounded resolution cutoff values remained identical at 3.7 Å. The reconstructed cryo-EM maps confirmed the gain in detail when motion correction is applied (Fig. 2E/F).

**Fig. 3:**
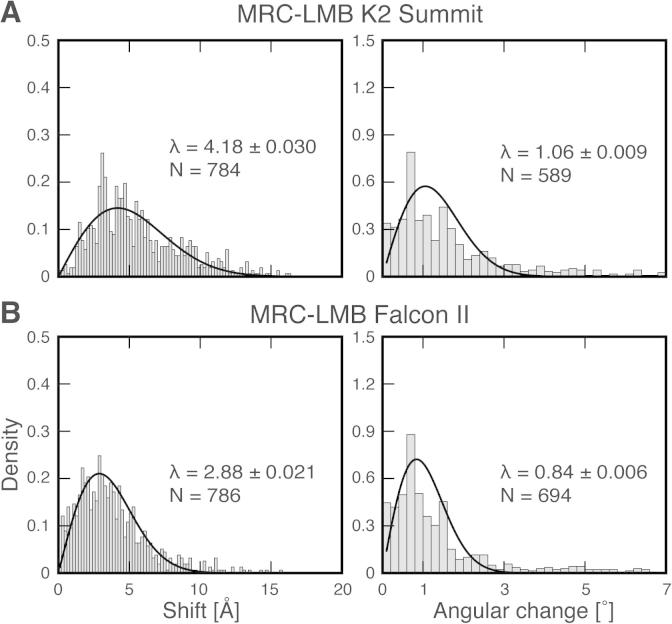
Comparison of the translational shift and angular change after ∼20 e^−^/Å^2^. The histograms of *x*–*y*-shifts (left) and the angular alignment change (right) are fitted with a Rayleigh distribution as described (Brilot et al., 2012). The maximum *λ* of each fit together with error and sample size is indicated in each graph. Histograms for the (A) MRC-LMB K2 Summit (2.0–19.5 e^−^/Å^2^) and (B) MRC-LMB Falcon II (2.4–19.1 e^−^/Å^2^) data sets are shown.

The optimal image acquisition conditions of the K2 Summit and Falcon II cameras require significantly different dose rates (Bai et al., 2013; Li et al., 2013b). Therefore, we compared the effect of the different exposure times on shift and rotation changes in the course of irradiating a total of 20 e^−^/Å^2^. For the K2 Summit and the Falcon II data sets, we fitted Rayleigh distributions to determine mean translational changes at 4.2 and 2.9 Å (Fig. 3). These shift distributions were broadened by an estimated translational alignment error of 0.8 and 0.9 Å, respectively (Table 1). The elevated shifts of the K2 data set are in line with the K2 Summit exposure being 4 times longer than the Falcon II, thus indicating a larger drift contribution. The fitted histograms of angular changes revealed a mean at 1.1° and 0.8° (Fig. 3). Thus, the measured rotations were close to the estimated angular error of 0.9° and 0.8°. In addition, the rotation distributions are broadened by the fact that changes in azimuthal angles can be compensated by a small translation of the TMV helix. This is also one of the reasons why observed rotations between 0 and 20 e^−^/Å^2^ are significantly larger than described in previous studies whereas the measured shifts are close to recent data on rotavirus (Campbell et al., 2012). In summary, beam-induced movement at low dose rates leads to larger shifts when compared with high-dose rates while rotations are hardly affected by the longer exposure.

### 2.3 The side-chain specific effect of radiation damage on the structure of TMV

**Fig. 4:**
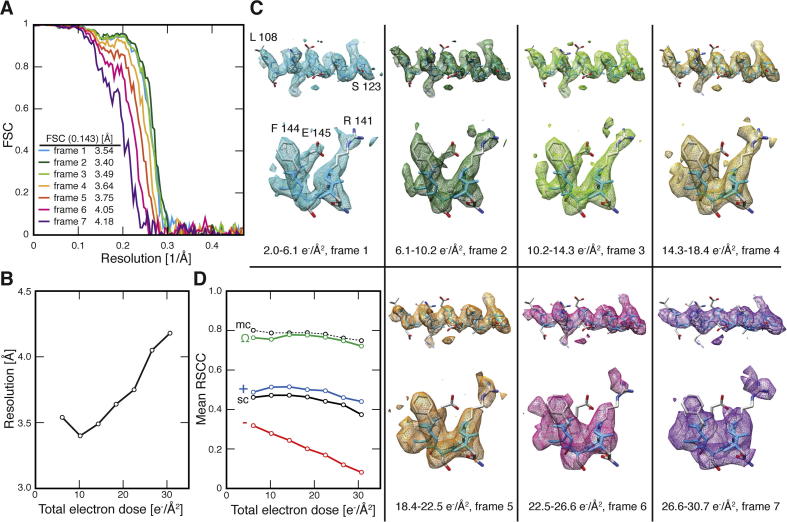
Movie of radiation damage effects on the high-resolution cryo-EM map. (A) Fourier shell correlation of seven 3D reconstructions from frame sets 1 to 7, from 6.1 to 30.7 e^−^/Å^2^. (B) Plot of the deposited dose vs. resolution at FSC cutoffs at 0.143. (C) Cryo-EM maps from frame sets 1 through 7 (EMD-2835 to EMD-2841). Details of helix LR (108–123) and residues 141–145 facing the outside surface of the helical rod. Side-chain densities of carboxyl residue E145 is preserved up to the 3rd frame set (14.3 e^−^/Å^2^) while positive residues are affected after frame set 5 and aromatic residues appear most stable up to the 7th frame set. (D) Average real-space cross-correlation (RSCC) of main-chain (mc) and side-chain (sc) residues as a function of deposited dose from 6.1 to 30.7 e^−^/Å^2^. The correlation drops faster for negative carboxyl (−) in comparison with positive (+) or aromatic (Ω) side chains.

In order to study the effect of radiation damage on the TMV structure, we processed TMV segments from individual frame sets of a large data set collected using the EMBL Falcon II. During the exposure a total of 30.7 e^−^/Å^2^ was deposited on the specimen and recorded in seven frame sets. According to the applied dose fractionation scheme, each presented frame set received a total of 4.1 e^−^/Å^2^. Using seven frame sets, we computed seven 3D reconstructions from ∼450,000 asymmetric units with determined resolutions ranging from 3.4 to 4.2 Å (Fig. 4A/B). The series of 3D reconstructions can be interpreted as time-resolved snapshots of the structure during exposure as radiation gets deposited on the specimen. Visually we observed the best-defined side-chain densities in the first three frame sets (Fig. 4C). It is important to note that the EMBL Falcon II data did not include the roll-in frame (corresponding to 56 ms or 2.0 e^−^/Å^2^ of exposure, see above). There is only a slight decrease in the quality of the first frame set map (2.0–6.1 e^−^/Å^2^) compared with the second frame set (6.1–10.2 e^−^/Å^2^) from 3.54 to 3.40 Å resolution, respectively.

In EM, the dose is generally expressed in the unit of e^−^/Å^2^ whereas the more general unit of radiation dose is measured in Grays (Joules per kilogram). In order to compare the effects of energy deposition to existing structural biology methods (Karuppasamy et al., 2011; Ravelli and Garman, 2006; Weik et al., 2000), we convert the electron dose as follows. The mean range of penetration of 300 kV is 840 μm for vitreous ice (ICRU, 1984). Following the calculation by Henderson (Henderson, 1990):

(1) $$\frac{4.1\;A{˚}^{-2}}{0.840\;\mathrm{mm}}\cdot 300\text{,}000\;\text{eV}=1.46\cdot 10^{23}\;\mathrm{eV}\;{\mathrm{mm}}^{-3}$$

(2) $$=2.3\cdot 10^{4}\;\text{J}\;{\text{cm}}^{-3}$$

(3) $$\approx 2.3\cdot 10^{7}\;\text{Grays}\left(\frac{\text{J}}{\text{kg}}\right)$$

at 300 kV voltage each frameset of 4.1 e^−^/Å^2^ corresponds to 2.3 × 10^7^ Grays.

As the dose increased beyond 14.3 e^−^/Å^2^ the first visible effects of radiation damage can be discerned in the map. In particular, negatively charged carboxyl side chains (e.g. E145 in Fig. 4C) quickly lost visible density from the third frame set onwards, while positively charged side chains followed at the 5th frame set (after 18.4 e^−^/Å^2^) (e.g. R141). Aromatic and aliphatic side chains appear more resistant to radiation damage than charged residues (e.g. F144). After 22.5 e^−^/Å^2^ the main chain of helix LR becomes less well defined. When quantitatively analyzing the impact of radiation damage on the structure by comparing the average real-space cross-correlation (RSCC) of main-chain versus side-chain atoms it is clear that both atom types were equally affected by radiation damage but the visibility of the main-chain specific deterioration appears weaker because of generally stronger densities (Fig. 4D). By contrast, the average RSCC of negatively charged residues decreased faster than that of positive or aromatic residues (Fig. 4D). In this case, the decline in real-space correlation is noticeable after deposition of 6.1 e^−^/Å^2^ or 3.5 × 10^7^ Grays. Beyond 18.4 e^−^/Å^2^ the density corresponding to side chains, but also the main chain, visibly lost detail. For an improved structural interpretation during *de novo* atomic model building, we found it beneficial to inspect the maps reconstructed from the first frame sets for details of carboxyl side-chain densities. Taken together it is clear that for the determination of sub-4 Å resolution structures it is critical to limit the dose to 20–25 e^−^/Å^2^ or 1.1–1.4 × 10^8^ Grays due to inferred radiation damage on the structure. This limited dose used here is 3 times higher than 4 × 10^7^ Gray that has been proposed as an experimental dose limit in X-ray crystallography (Owen et al., 2006). Alternatively, more recent approaches have down-weighted the later stages of the exposure to limit radiation damage effects (Lu et al., 2014; Scheres, 2014; Wang et al., 2014).

### 2.4 High-resolution 3D reconstruction

**Fig. 5:**
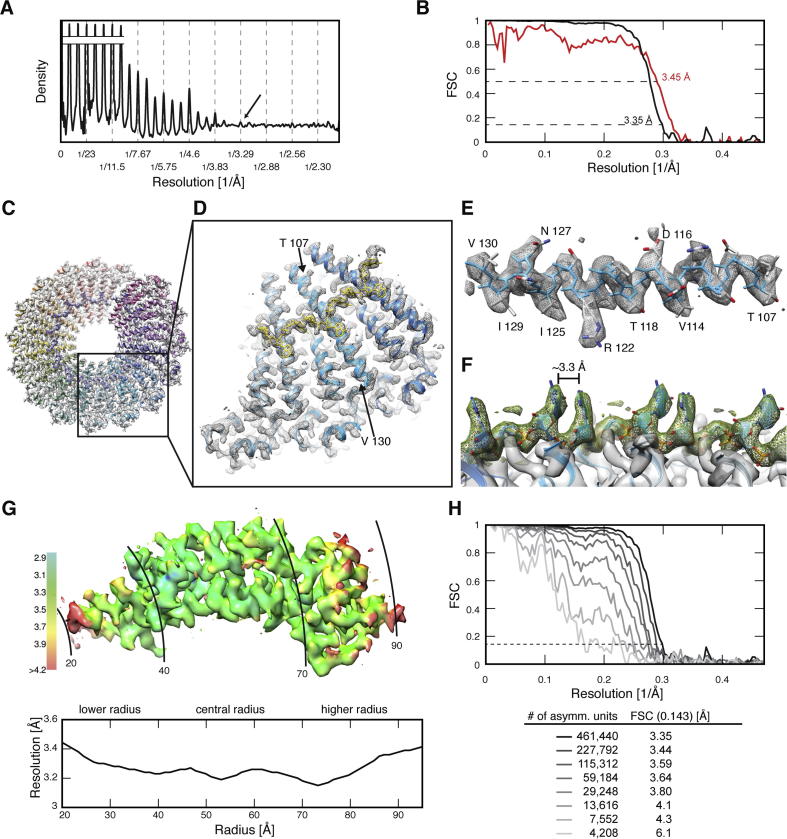
High-resolution cryo-EM reconstruction of TMV at 3.35 Å resolution. (A) Collapsed power spectrum averaged from 8336 segments recorded on a Falcon II camera. (B) Fourier shell correlation (FSC) of half-set 3D reconstructions (black) compared with the FSC of the 3D reconstruction and the simulated map of the refined PDB model (red, PDB code: 4udv). (C) Cross section through TMV rod including 16 subunits (EMD-2842). (D) Inset of three adjacent subunits as displayed in other Figures throughout the manuscript. (E) Isolated helix LR with visible intermediately sized side chains of I129, N127, I125 and V114. (F) Density of six RNA bases stacked to pair with a separating distance of approx. 3.3 Å. (G) Top. TMV map superimposed at corresponding radial position with a color-coded local resolution measurement using ResMap (Kucukelbir et al., 2014). Bottom. Radial resolution distribution as measured by the 0.143 FSC cutoff from a sliding 20 Å-wide cylindrical mask. (H) Eight Fourier shell correlations and respective cutoff values with increasing number of asymmetric units.

In order to obtain the highest-resolution map, we further processed a significantly larger data set. For this analysis, we focused on the available in-house Falcon II camera at the EMBL. We expect that similar results could be obtained with a K2 Summit camera as a number of high-resolution structures acquired on K2 cameras have been reported (Li et al., 2013a; Liao et al., 2013; Lu et al., 2014). First, we analyzed the sum of the in-plane rotated power spectra from overlapping segments of 806 viruses (Table 2). From the collapsed power spectrum it is apparent that a peak from the meridional layer line at 1/3.29 Å is still detectable (Fig. 5A). Therefore, we reconstructed the cryo-EM structure to a resolution of 3.35 Å by limiting the dose to an optimum (see above) of 18.4 e^−^/Å^2^ using the described segment-based motion correction scheme (see above) (Fig. 5B). The FSC computed between the final 3D reconstruction and the simulated map of the refined PDB structure gave a similar resolution criterion at 3.45 Å at the FSC 0.5 cutoff (Rosenthal and Henderson, 2003) (Fig. 5B). In this case, the detectable layer-line peaks appear to be representative of the obtained resolution after image processing. At this resolution, the LR helix reveals a well resolved α-helical main-chain path including intermediately sized side chains such as I129, N127, I125 and V114. (Fig. 5C–E). In addition, high-resolution density features of the RNA that is packed between two helical turns of the TMV coat protein subunits are in agreement with the resolution claim (Fig. 5F).

As RNA bases stack between adjacent TMV subunits and are well separated by a distance of approx. 3.3 Å (Fig. 5F), the cryo-EM reconstruction suggests that some regions have a resolution better than 3.35 Å as obtained from the overall FSC 0.143 cutoff. To further investigate this observation, we measured the local resolution of the map by sliding a 20 Å wide cylindrical mask towards increasing radius (Fig. 5G, bottom). The highest measured resolution of 3.2 Å is located at 40, 55 and 75 Å radial position where the RNA chain and a cluster of aromatic side-chains reside. Furthermore, we used the program ResMap (Kucukelbir et al., 2014) to assess local resolution in the map to confirm this trend (Fig. 5G, top).

Finally, we computed eight 3D reconstructions from increasing numbers of randomly selected viruses, in order to investigate the relationship between number of asymmetric units and attainable resolution. First, we found that as few as 7100 asymmetric units were sufficient to compute a near-atomic resolution structure. Second, in our tested range between ∼4000 and ∼450,000 asymmetric units the addition of more data constantly improved the resolution of the reconstructed maps (Fig. 5H). In comparison with previous TMV reconstructions from film micrographs where 200,000 asymmetric were required to compute a 4.7 Å structure (Sachse et al., 2007), cryo-micrographs recorded on DEDs required an order of magnitude fewer asymmetric units to achieve a comparable resolution. Beyond 4 Å or ∼30,000 asymmetric units, doubling the data results in an improvement of 0.1–0.2 Å up to the maximum resolution of 3.35 Å. The exact relationship is determined by the B-factor of the 3D reconstructions (Rosenthal and Henderson, 2003). The observed continuous improvement suggests that adding more virus particles to the data set could still help to reach slightly higher resolution, although in a previous study it was shown that beyond ∼700,000 asymmetric units, adding more data did not improve the resolution beyond 3.3 Å of the respective reconstructions (Li et al., 2013a).

## 3 Discussion

Using new DEDs, we were able to reconstruct near-atomic resolution structures of TMV from the same electron microscope recorded on the K2 Summit and Falcon II at 4.0 and 3.7 Å resolution with a total of 44,000 asymmetric units. We conclude that both detectors are capable of generating high-resolution image reconstructions. As optimal operation of the K2 summit requires 4–5 times longer exposure times when compared with the Falcon II, drift will have a stronger influence on the transfer of high-resolution image contrast. Therefore, the slightly lower resolution obtained using the K2 Summit may be attributed to a longer exposure time used for each frame. The detector’s performance proved comparable in the present study, which is in line with previous analysis based on the measurement of DQE and MTF curves (McMullan et al., 2014). This is somewhat in contrast to another study (Ruskin et al., 2013) comparing the two cameras at voltages of 200 kV, where the K2 Summit appeared to perform better than the Falcon II. Overall, large samples such as TMV may benefit from the improved high-resolution transfer of the Falcon II, whereas smaller samples would likely be easier to detect and align on K2 Summit micrographs due to the improved low-resolution contrast.

The ability to record electron cryo-micrographs in ‘movie mode’ has opened up new opportunities to compensate for motion during the exposure. Here, we compared the performance between segment-based and the micrograph-based motion correction approach. Comparison of the two types of corrections is useful in particular for smaller structures where segment or particle-based motion correction is more error prone and tracking of the particle within frame sets is not possible. We see an improvement in the resolution of 3D reconstructions from 4.0 to 3.7 Å by compensating for motion correction with both segment-based and micrograph-based strategies. While the segment-based FSC resolution curves show slightly higher correlations, the micrograph-based correction has the practical advantage that it is significantly computationally less expensive as it can be dealt with prior to the refinement procedure. By contrast, segment-based motion correction effectively increases the computational cost by the number of frame sets being processed. In addition to practical considerations, the micrograph-based motion correction is less dependent on the type of imaged structures, while segment-based correction is only beneficial when maximum resolution is required. With better algorithms for particle-based motion correction of small specimens one can compare their movement with large TMV particles and conclude whether particle or micrograph-based motion correction or a combination of the two provide optimal results.

To investigate the effect of radiation damage on the TMV structure, we used individual frame sets to reconstruct a series of cryo-EM maps from 450,000 asymmetric units to visualize the impact of accumulated radiation on the structure of TMV. Our TMV reconstructions determined between 3.4 and 4.2 Å resolution show that at a voltage of 300 kV and beyond a dose of 18.4 e^−^/Å^2^ there is little high-resolution structural data that will contribute to improving the cryo-EM map beyond 4 Å. This is in agreement with previous sub-4 Å structures, where the dose was either restricted (Li et al., 2013a; Liao et al., 2013) or dose weighting was applied (Lu et al., 2014; Wang et al., 2014). Nevertheless, the precise impact of radiation damage is dependent on the chemical composition and the dimensions of the visualized molecule. We demonstrated that radiation affects the molecular structure specifically with the first impact on carboxylate residues, followed by other large and intermediate size side chains. The specific nature of radiation damage is in agreement with a recent side-chain analysis (Allegretti et al., 2014) and dose series observations (Bartesaghi et al., 2014) of high-resolution cryo-EM structures recorded with DEDs. As a result, the local damage and destabilization of such parts of the structure also deteriorate the resolution of the main chain. These qualitative observations are in agreement with a number of previous near-atomic cryo-EM maps obtained from film (Grigorieff et al., 1996; Sachse et al., 2007). Interestingly, very similar side-chain specific effects on protein structure such as decarboxylation were observed in X-ray diffraction experiments on acetylcholinesterase and lysozyme (Weik et al., 2000). These results together with our observations support previous propositions that both electrons and X-rays cause radiation damage by the same chemical mechanisms initiated via free radicals and subsequent chemical reactions (Henderson, 1990).

Finally, we determined the TMV structure at 3.35 Å resolution using electron cryo-micrographs. It is interesting to note that unprocessed data from the sum of in-plane rotated power spectra revealed a peak at very similar resolution of 1/3.29 Å. Local resolution measures and map details show that RNA and aromatic side-chain features exceed the average resolution cutoff up to 3.2 Å. Using electron diffraction, Kühlbrandt and co-workers, showed that oriented TMV rafts diffracted to measurable 2.3 Å (Cyrklaff and Kühlbrandt, 1994). This resolution discrepancy indicates that the imaging process of biological specimens in the electron microscope is currently not capable of recovering this kind of order in a biological object. The highest resolution image reconstruction reported was recently obtained from images of the mitochondrial ribosome that has a large content of RNA at 3.2 Å resolution (Amunts et al., 2014). A number of optical limitations have been discussed (Zhang and Zhou, 2011). While beam tilt can limit the optical resolution, the micrographs were taken on a Titan Krios microscope set up with an electron beam in parallel illumination due to its additional C3 lens. Furthermore, the curvature of the Ewald sphere and the resulting defocus gradient across large particles are known to limit the resolution. But at a voltage of 300 kV and with TMV as a relatively thin specimen, the effects will only become limiting significantly below a resolution of 3 Å. Other factors limiting the accuracy of the 3D reconstruction include image-processing steps, such as the precise contrast-transfer-function (CTF) determination and alignment. While the new capabilities of DEDs have helped to elucidate and better understand the interaction of the electron beam with the specimen and the induced motion, more details may be required to extract a near-perfect still image required for the optimal 3D reconstruction. In the future, further improvements in sample support, detector technology and additional microscope hardware can be expected to contribute to our understanding of the current resolution limitations.

There is no doubt that the use of the new DED hardware represents a leap in quality improvement over previous 3D image reconstructions recorded with conventional detector systems. From these data it is becoming increasingly clear that in the future the molecular weight threshold of molecules that can be routinely visualized at high resolution will decrease to significantly smaller than 500 kDa (Liao et al., 2013; Lu et al., 2014; Vinothkumar et al., 2014), which will make the cryo-EM technique even more attractive to the structural biology community. It will be interesting to explore the boundaries of smaller macromolecules of 100 s of kDa in order to determine their structures to sub-nanometer detail and thus making an entirely new repertoire of proteins amenable to the cryo-EM technique. We expect that a combination of innovative data collection strategies and software solutions will be required to maximize the output from DEDs. In agreement with a number of other articles, the current manuscript demonstrates that atomic resolution close to 3 Å is feasible for large macromolecular complexes with DED technology. Nevertheless, as shown for the highly ordered TMV specimen it is currently not possible to reach sub-3 Å resolution. Therefore, more studies are required to explore the relative contributions of optics, specimen interaction and detector shortcomings to the current limitations.

## 4 Materials and methods

### 4.1 Virus preparation

Virus was grown in *Nicotiana tabacum* and harvested infected plants stored at −20 °C. After mincing, the leaf slurry was liquidized in a Vortex mixer to reduce the fibrous nature of the plant remains. The homogenate was then filtered and spun (12,000×*g* for 30 min) to pellet the particulate matter. Each filtrate was precipitated in 30% (w/v) PEG 6000, followed by 2.5 ml 5 M NaCl washes. The precipitate was recovered by centrifugation at 12,000×*g* for 10 min and re-extracted 3 times with 10 mM Na_2_HPO_4_/0.1% (w/v) ascorbic acid. The TMV was pelleted at 50,000 rev/min for 30 min and the dark green layer washed off the top of the glass like pellets. The pellets were then left under Na phosphate buffer (*I* = 0.1 M, pH 7.0) at 4 °C overnight to soften, before being re-suspended until the TMV solution clarified. Differential centrifugation was repeated until a clean preparation was obtained.

### 4.2 Electron microscopy

First, we prepared EM samples and recorded a data set at the MRC-LMB in the following way: a total of 2.5 μl of 5.5 mg/ml TMV solution was applied to Quantifoil 2/2 Cu 300 mesh grids. Subsequently, the grids were mounted in the Vitrobot Mark 4 with a ∼10 s time between application and blotting. The Vitrobot blotted with force of −15, for a total of 2.5 s with a 0.5 s drain time at 100% humidity at 10 °C. The MRC-LMB FEI Titan Krios was operated at 300 kV with an extraction voltage of 3950 V (gun lens 3). For Falcon II imaging, an 800-nm diameter electron beam was used with a 70 μm C2 aperture. Spot size 4 was used in Nanoprobe mode at a nominal magnification of 75,000 (calibrated pixel size 1.06 Å), leading to a dose rate at the camera level of ∼50 e^−^/pixel/s. An exposure time of one second was set accumulating a total dose of 43 e^−^/Å^2^ in the integrated image (16 frame sets were processed while 18 were collected including the roll-in and roll-out frames that were discarded). Falcon II data at the MRC-LMB was available in 16 frame sets using the home-built software solution. Falcon II micrographs were recorded with an under-focus between 1.0 and 3.0 μm. For K2 imaging, the same grid was imaged in the same microscopy session. Data was collected in EFTEM mode at a nominal magnification of 105,000 (calibrated pixel size 1.13 Å) with an 800-nm beam at spot size 6, corresponding to a dose rate at the camera level of 7 e^−^/pixel/s. The applied dose on the sample was estimated using known dose response curves for both detectors. Other hardware settings of the microscope were kept the same. An exposure time of 4.4 s was set accumulating a total dose of 43 e^−^/Å^2^ in the integrated image (22 frames recorded over the entire movie). Images were collected with an under-focus of 1.2 and 2.5 μm in super-resolution mode of the K2 camera (pixel size 0.56 Å) using SerialEM (Mastronarde, 2005) with the energy slit of the Gatan Quantum energy filter retracted. Subsequently, all K2 micrographs were binned 2 × 2 by windowing in Fourier space.

Second, we describe EM sample preparation and data collection performed at EMBL: a total of 2.5 μl of 11 mg/ml TMV solution was applied on Quantifoil 2/2 200 mesh grid under the light microscope to ensure proper application and spreading. Subsequently, the grids were mounted in the Vitrobot Mark 3 with a ∼30 to 45 s time between application and blotting. The Vitrobot blotted with an offset of −2 mm, for a total of 8 s without drain time at 90% humidity. The EMBL FEI Titan Krios was operated at 300 kV with an extraction voltage of 4100 V (gun lens 3). The 600-nm diameter electron beam was aligned with the 70 μm C2 aperture using a spot size 6 in Nanoprobe mode. The micrographs were recorded at a nominal magnification of 75,000 giving a final pixel size of 1.06 Å on the specimen at an under-focus between 1.0 and 4.5 μm. The data was acquired in a fully automated manner with FEI EPU software using a 3 × 3 image matrix over a 2 μm hole, which includes beam shifts up to 800 nm away from the optical axis. We set the exposure time to 0.836 s accumulating a total of 30.7 e^−^/Å^2^ measured dose in the integrated image (including the roll-in frame). The frames were merged into even seven frame sets from 2.0 to 30.7 e^−^/Å^2^ (excluding roll-in and roll-out frame) using FEI’s integrated 7-frame bin solution.

### 4.3 Image processing

All of the described cryo-EM maps were generated using the standardized procedure of SPRING (Desfosses et al., 2014), except for the micrograph-based motion correction that was performed using MOTIONCORR (Li et al., 2013a). In summary, SPRING is a comprehensive single-particle based helical reconstruction package that was originally inspired by the IHRSR approach (Egelman, 2000). Briefly, the CTF parameters were determined using MICCTFDETERMINE that relies on CTFFIND with subsequent CTFTILT measurements (Mindell and Grigorieff, 2003). We used the diagnostic output of MICCTFDETERMINE to discard micrographs that did not show Thon rings exceeding 6 Å. 80% were excluded because they were either empty or too crowded with TMV and 12% based on relatively poor Thon rings. 8% of the micrographs had a good density of viruses and showed Thon rings. After the selection step, we cropped TMV particles using E2HELIXBOXER from EMAN2 (Tang et al., 2007). A stack of overlapping segments of a size of 350 × 350 Å with the segment-specific CTF convolved was produced using SEGMENT with a step size of 90 Å. The segments were subjected to 20 rounds of iterative refinement using SEGMENTREFINE3D from low-resolution to maximum-resolution target using the standard refinement strategies of SPRING. In order to avoid over-fitting of noise during alignment, we restricted the alignment search to segments low-pass filtered to 11 Å. This approach yields identical FSC curves from independent half-set refinements (Scheres and Chen, 2012). The segment-based motion correction was also implemented in SPRING. First, the sum of the frame sets for each micrograph was subjected up to maximum resolution refinement using SEGMENTREFINE3D. Second, a new stack containing the corresponding frames of each segment was generated using SEGMENT. The previously determined orientation parameters were used as starting parameters for four subsequent local refinement cycles at maximum resolution (restrained *x*- and *y*-search ±7 and ±4 Å, restrained angular search ±2°) with SEGMENTREFINE3D. The displayed densities were generated using SEGREFINE3DINSPECT with an applied sharpening B-factor of −150 1/Å^2^ and the corresponding 0.143 resolution cutoff (Rosenthal and Henderson, 2003). Translational and angular alignment errors were estimated using the forward difference of measurements along a helix as described previously (Sachse et al., 2007) where a small fraction of segments with distances and angles larger than 5 Å or 5° were excluded as they failed to align (Table 1).

### 4.4 Atomic coordinate refinement

We performed a real-space coordinate refinement in order to improve the recent PDB structure (Sachse et al., 2007) using the 3.35 Å resolution cryo-EM map. Initially, one of the 2OM3 conformers was placed into the EM density using the rigid-body fit option as implemented in Chimera (Pettersen et al., 2004) and an oligomer of nine neighboring TMV subunits (3 × 3) was generated using the helical symmetry of the virus. A series of in-house scripts based on CCP4 and cctbx/PHENIX functions (Adams et al., 2010; Winn et al., 2011) was employed to streamline subsequent refinement and validation of the coordinate models. A map segment corresponding to the 9-mer was carved from the reconstructed map using a mask encompassing all grid points including and extending 3.5 Å outwards of the model coordinates (including RNA). The segmented density was centered in a cubic box of 180 × 180 × 180 voxels. Likewise, the rigid-body fitted starting model was centered in a cubic unit cell of P1 symmetry with a cell edge of 191.16 Å (=180 × pixel size) to ensure uniform grid sampling of experimental and computed model density maps. A uniform isotropic B-factor of 50 1/Å^2^ was assigned to all atoms and not further refined. Subsequently, the model was subjected to five cycles of geometry-restrained real-space refinement by gradient-driven minimization of a combined map and restraint target as implemented in PHENIX/cctbx (Adams et al., 2010). A local grid search to correct side-chains with incorrect rotamer assignments or poor density correlation was iterated with global optimization aimed at improvement of the overall density fit. Weights on density map and geometry restraints were optimized during each refinement cycle. In the current implementation, we did not permit more than one conformer per residue. The central subunit of the resulting model was inspected and corrected by manual model building in Coot (Emsley and Cowtan, 2004) and the modified 9-mer was subsequently subjected to three additional cycles of geometry-restrained real-space refinement. The progress of refinement was evaluated by computing the real-space cross-correlation (RSCC) for each residue along the monomer chain. To determine the overall agreement of the refined coordinate model with the observed data, refinements were run at different target resolutions. Finally, we used a target resolution of 3.2 Å because those refined atomic coordinates showed good agreement with the map and best geometry statistics. The Fourier shell correlation (FSC) at the 0.5 criterion between the map computed from the refined model at each target resolution and the experimental map filtered at the expected maximum resolution was used to assess the possibility of over-fitting (Chen et al., 2013). Finally, we re-expanded the central TMV subunit coordinates into a helix of the same dimension as the 3D reconstruction, simulated a noise-free map at 2 Å resolution and computed the FSC with the determined 3D reconstruction, which gave a resolution of 3.45 Å at the 0.5 criterion (Fig. 5B). The figures of the manuscript were prepared using UCSF Chimera (Pettersen et al., 2004).

*We deposited the EM maps from the K2-LMB and FalconII-LMB data sets at the EM Data Bank (EMDB-2833 and EMDB-2834). The EM densities corresponding to the radiation damage series are EMDB-2835, EMDB-2836, EMDB-2837, EMDB-2838, EMDB-2839, EMDB-2840, EMDB-2841. The 3.35 Å map recorded using the FalconII-EMBL data set is available as EMDB-2842. The refined atomic coordinates have accession code PDB-4udv at the Protein Data Bank. The software SPRING includes the used segment-based frame processing and is available from the author’s website at* http://www.sachse.embl.de/emspring.

## Author contributions

S.A.F., T.A.M.B. and C.S. designed the research. C.S. purified TMV. W.J.H.H. and T.A.M.B. prepared cryo-EM samples and collected micrographs. S.A.F. and C.S. processed cryo-EM images. S.A.F. and A.J.J. refined the atomic coordinates. S.A.F. and C.S. wrote the paper with support from all other authors.

## Figures and Tables

**Table 1:** Alignment error estimates of processed data sets.

	K2 LMB			Falcon II LMB			Falcon II EMBL		
	*x*-Shift [Å]	Out of plane [°]	In plane [°]	*x*-Shift [Å]	Out of plane [°]	In plane [°]	*x*-Shift [Å]	Out of plane [°]	In plane [°]
Sum (no motion correction)	0.7	0.8	0.2	0.7	0.9	0.3	0.9	0.7	0.2
Frames (segment-based motion correction)	0.9	1.0	0.4	0.8	1.0	0.4	0.9	0.8	0.3

**Table 2:** Summary of processed data sets.

Data set	01: K2, MRC-LMB Cambridge	02: Falcon II, MRC-LMB Cambridge	03: Falcon II, EMBL Heidelberg
Microscope	FEI Titan Krios	FEI Titan Krios	FEI Titan Krios
Camera	Gatan K2 Summit	FEI Falcon II	FEI Falcon II
Included (total) dose [e^−^/Å^2^]	21.5 (43)	19.1 (43)	16.4 (30.7)
Exposure time [s]	4.4	1.0	0.84
Included (total) micrographs	14 (14)	28 (53)	109 (289)
# Of viruses/segments	69/784	72/786	806/8336
# Of asymmetric units	43,904	43,648	449,280

FSC (0.143) [Å]			
No corrections/total dose	4.6	4.2	3.49
No correction/dose limitation	4.4	4.0	3.35
Motion correction/dose limitation	4.0	3.7	3.35

Figure	1, 3	1, 2, 3	2, 4, 5

## References

[b0005] Adams P.D., Afonine P.V., Bunkoczi G., Chen V.B., Davis I.W., Echols N., Headd J.J., Hung L.W., Kapral G.J., Grosse-Kunstleve R.W. (2010). PHENIX: a comprehensive python-based system for macromolecular structure solution. Acta Crystallogr..

[b0010] Adrian M., Dubochet J., Lepault J., McDowall A. (1984). Cryo-electron microscopy of viruses. Nature.

[b0015] Allegretti M., Mills D.J., McMullan G., Kühlbrandt W., Vonck J. (2014). Atomic model of the F420-reducing [NiFe] hydrogenase by electron cryo-microscopy using a direct electron detector. eLife.

[b0020] Amunts A., Brown A., Bai X.C., Llacer J.L., Hussain T., Emsley P., Long F., Murshudov G., Scheres S.H.W., Ramakrishnan V. (2014). Structure of the yeast mitochondrial large ribosomal subunit. Science.

[b0025] Bai X.-C., Fernandez I.S., McMullan G., Scheres S.H. (2013). Ribosome structures to near-atomic resolution from thirty thousand cryo-EM particles. eLife.

[b0030] Bartesaghi A., Matthies D., Banerjee S., Merk A., Subramaniam S. (2014). Structure of β-galactosidase at 3.2-Å resolution obtained by cryo-electron microscopy. Proc. Natl. Acad. Sci. U.S.A..

[b0035] Bernal J.D., Fankuchen I. (1941). X-ray and crystallographic studies of plant virus preparations. III. J. Gen. Physiol..

[b0040] Brilot A.F., Chen J.Z., Cheng A., Pan J., Harrison S.C., Potter C.S., Carragher B., Henderson R., Grigorieff N. (2012). Beam-induced motion of vitrified specimen on holey carbon film. J. Struct. Biol..

[b0045] Campbell M.G., Cheng A., Brilot A.F., Moeller A., Lyumkis D., Veesler D., Pan J., Harrison S.C., Potter C.S., Carragher B. (2012). Movies of ice-embedded particles enhance resolution in electron cryo-microscopy. Structure.

[b0050] Chen S., McMullan G., Faruqi A.R., Murshudov G.N., Short J.M., Scheres S.H.W., Henderson R. (2013). Ultramicroscopy.

[b0055] Clare D.K., Orlova E.V. (2009). 4.6 Å Cryo-EM reconstruction of tobacco mosaic virus from images recorded at 300 keV on a 4k × 4k CCD camera. J. Struct. Biol..

[b0060] Cyrklaff M., Kühlbrandt W. (1994). High-resolution electron microscopy of biological specimens in cubic ice. Ultramicroscopy.

[b0065] Desfosses A., Ciuffa R., Gutsche I., Sachse C. (2014). SPRING – an image processing package for single-particle based helical reconstruction from electron cryomicrographs. J. Struct. Biol..

[b0070] Egelman E.H. (2000). A robust algorithm for the reconstruction of helical filaments using single-particle methods. Ultramicroscopy.

[b0075] Emsley P., Cowtan K. (2004). Coot: model-building tools for molecular graphics. Acta Crystallogr. D Biol. Crystallogr..

[b0080] Faruqi A.R., Henderson R. (2007). Electronic detectors for electron microscopy. Curr. Opin. Struct. Biol..

[b0085] Fernandez I.S., Bai X.-C., Hussain T., Kelley A.C., Lorsch J.R., Ramakrishnan V., Scheres S.H.W. (2013). Molecular architecture of a eukaryotic translational initiation complex. Science.

[b0090] Ge P., Zhou Z.H. (2011). Hydrogen-bonding networks and RNA bases revealed by cryo electron microscopy suggest a triggering mechanism for calcium switches. Proc. Natl. Acad. Sci. U.S.A..

[b0095] Grigorieff N. (2013). Direct detection pays off for electron cryo-microscopy. eLife.

[b0100] Grigorieff N., Ceska T., Downing K., Baldwin J., Henderson R. (1996). Electron-crystallographic refinement of the structure of bacteriorhodopsin. J. Mol. Biol..

[b0105] Henderson R. (1990). Cryo-protection of protein crystals against radiation damage in electron and X-ray diffraction. Proc. R. Soc. Lond. B Biol. Sci..

[b0110] Henderson R., Baldwin J.M., Ceska T.A., Zemlin F., Beckmann E., Downing K.H. (1990). Model for the structure of bacteriorhodopsin based on high-resolution electron cryo-microscopy. J. Mol. Biol..

[b0115] ICRU (1984). Stopping Powers for Electrons and Positrons (Report 37) (Bethesda, Maryland, USA)

[b0120] Karuppasamy M., Karimi Nejadasl F., Vulovic M., Koster A.J., Ravelli R.B.G. (2011). Radiation damage. J. Synchrotron Radiat..

[b0125] Kucukelbir A., Sigworth F.J., Tagare H.D. (2014). Quantifying the local resolution of cryo-EM density maps. Nat. Methods.

[b0130] Li X., Mooney P., Zheng S., Booth C.R., Braunfeld M.B., Gubbens S., Agard D.A., Cheng Y. (2013). Electron counting and beam-induced motion correction enable near-atomic-resolution single-particle cryo-EM. Nat. Methods.

[b0135] Li X., Zheng S.Q., Egami K., Agard D.A., Cheng Y. (2013). J. Struct. Biol..

[b0140] Liao M., Cao E., Julius D., Cheng Y. (2013). Structure of the TRPV1 ion channel determined by electron cryo-microscopy. Nature.

[b0145] Lu P., Bai X.-C., Ma D., Xie T., Yan C., Sun L., Yang G., Zhao Y., Zhou R., Scheres S.H.W. (2014). Three-dimensional structure of human γ-secretase. Nature.

[b0150] Mastronarde D.N. (2005). Automated electron microscope tomography using robust prediction of specimen movements. J. Struct. Biol..

[b0155] McMullan G., Faruqi A.R., Clare D., Henderson R. (2014). Comparison of optimal performance at 300 keV of three direct electron detectors for use in low dose electron microscopy. Ultramicroscopy.

[b0160] Mindell J.A., Grigorieff N. (2003). Accurate determination of local defocus and specimen tilt in electron microscopy. J. Struct. Biol..

[b0165] Owen R.L., Rudiño-Piñera E., Garman E.F. (2006). Experimental determination of the radiation dose limit for cryocooled protein crystals. Proc. Natl. Acad. Sci. U.S.A..

[b0170] Pettersen E.F., Goddard T.D., Huang C.C., Couch G.S., Greenblatt D.M., Meng E.C., Ferrin T.E. (2004). UCSF Chimera – a visualization system for exploratory research and analysis. J. Comput. Chem..

[b0175] Ravelli R., Garman E. (2006). Radiation damage in macromolecular cryocrystallography. Curr. Opin. Struct. Biol..

[b0180] Rosenthal P.B., Henderson R. (2003). Optimal determination of particle orientation, absolute hand, and contrast loss in single-particle electron cryomicroscopy. J. Mol. Biol..

[b0185] Ruskin R.S., Yu Z., Grigorieff N. (2013). Quantitative characterization of electron detectors for transmission electron microscopy. J. Struct. Biol..

[b0190] Sachse C., Chen J.Z., Coureux P.-D., Stroupe M.E., Fändrich M., Grigorieff N. (2007). High-resolution electron microscopy of helical specimens: a fresh look at tobacco mosaic virus. J. Mol. Biol..

[b0195] Scheres S.H. (2014). Beam-induced motion correction for sub-megadalton cryo-EM particles. eLife.

[b0200] Scheres S.H.W., Chen S. (2012). Prevention of overfitting in cryo-EM structure determination. Nat. Methods.

[b0205] Shigematsu H., Sigworth F.J. (2013). Noise models and cryo-EM drift correction with a direct-electron camera. Ultramicroscopy.

[b0210] Tang G., Peng L., Baldwin P.R., Mann D.S., Jiang W., Rees I., Ludtke S.J. (2007). EMAN2: an extensible image processing suite for electron microscopy. J. Struct. Biol..

[b0215] Unwin N. (2005). Refined structure of the nicotinic acetylcholine receptor at 4 A resolution. J. Mol. Biol..

[b0220] Vinothkumar K.R., McMullan G., Henderson R. (2014). Molecular mechanism of antibody-mediated activation of β-galactosidase. Structure.

[b0225] Voorhees R.M., Fernandez I.S., Scheres S.H.W., Hegde R.S. (2014). Structure of the Mammalian ribosome-sec61 complex to 3.4 Å resolution. Cell.

[b0230] Wang Z., Hryc C.F., Bammes B., Afonine P.V., Jakana J., Chen D.-H., Liu X., Baker M.L., Kao C., Ludtke S.J. (2014). An atomic model of brome mosaic virus using direct electron detection and real-space optimization. Nat. Commun..

[b0235] Weik M., Ravelli R.B., Kryger G., McSweeney S., Raves M.L., Harel M., Gros P., Silman I., Kroon J., Sussman J.L. (2000). Specific chemical and structural damage to proteins produced by synchrotron radiation. Proc. Natl. Acad. Sci. U.S.A..

[b0240] Winn M.D., Ballard C.C., Cowtan K.D., Dodson E.J., Emsley P., Evans P.R., Keegan R.M., Krissinel E.B., Leslie A.G.W., McCoy A. (2011). Overview of the CCP4 suite and current developments. Acta Crystallogr. D Biol. Crystallogr..

[b0245] Yu X., Jin L., Zhou Z.H. (2008). 3.88 A structure of cytoplasmic polyhedrosis virus by cryo-electron microscopy. Nature.

[b0250] Zhang X., Zhou Z.H. (2011). Limiting factors in atomic resolution cryo electron microscopy: no simple tricks. J. Struct. Biol..

[b0255] Zhang X., Settembre E., Xu C., Dormitzer P.R., Bellamy R., Harrison S.C., Grigorieff N. (2008). Near-atomic resolution using electron cryomicroscopy and single-particle reconstruction. Proc. Natl. Acad. Sci. U.S.A..

